# ﻿Phylogenetic relationships among western Atlantic representatives of *Pilumnus* Leach, 1816 (Decapoda, Brachyura, Pilumnidae) based on molecular markers, with comments on biogeography

**DOI:** 10.3897/zookeys.1253.160114

**Published:** 2025-09-25

**Authors:** Lucas Oliveira-Rogeri, Tatiana Magalhães, Darryl L. Felder, Fernando L. Mantelatto

**Affiliations:** 1 Laboratory of Bioecology and Crustacean Systematics (LBSC), Department of Biology, Faculty of Philosophy, Science and Letters at Ribeirão Preto (FFCLRP), University of São Paulo (USP), Ribeirão Preto, SP, Brazil University of São Paulo Ribeirão Preto Brazil; 2 Department of Biology and Laboratory for Crustacean Research, Department of Biology, University of Louisiana at Lafayette, Lafayette, Louisiana, 70504, USA University of Louisiana at Lafayette Lafayette United States of America; 3 Department of Invertebrate Zoology, National Museum of Natural History, Smithsonian Institution, Washington, DC, 20560, USA National Museum of Natural History, Smithsonian Institution Washington United States of America

**Keywords:** America, biodiversity, genetic distance, hairy crabs, phylogeny, species delimitation

## Abstract

*Pilumnus* is a highly speciose brachyuran crab genus distributed in tropical and temperate oceans. Previous systematic studies of the genus have revealed a high degree of uncertainty as to phylogenetic relationships. This is particularly evident among western Atlantic representatives, which have never been examined comprehensively from a phylogenetic perspective. A comparative molecular phylogenetic analysis of *Pilumnus* from the western Atlantic was conducted using mitochondrial sequences of the 16S rRNA and Cytochrome Oxidase subunit I genes. Sequences for 14 species from the western Atlantic were generated, included in the analyses, and compared to sequences of congeners from other biogeographic regions. Phylogenetic hypotheses including divergence time data were derived, genetic distances were calculated, and species delimitation methods were applied. Our results reveal that these taxa represent two distinct lineages that do not reflect modern geography in their distributions. The phylogenetic reconstruction identified two separate lineages presently treated under *P. reticulatus*, and species delimitation methods suggested that they may correspond to two separate species. Similarly, delimitation analyses indicated that an accessed specimen phylogenetically close to *P. lacteus* may represent an undescribed species. The results evince the importance of molecular phylogenetic studies in comprehending the evolutionary and biogeographic patterns that characterise the marine decapod biota.

## ﻿Introduction

Bristle crabs of the genus *Pilumnus* Leach, 1816 are a speciose group of brachyurans distributed worldwide. Species of this genus are common components of coastal and shallow water ecosystems such as coral reefs, rocky bottoms, mangrove roots, and pilings or pillars of piers, breakwaters, and docks (Moraes et al. 2015; Fahimi and Naderloo 2023). Fahimi et al. (2021) defined the group as a “catch-all” due to the morphological heterogeneity found among its members. The present systematics and taxonomy of the group suggest no clear phylogenetic relationships within the genus (Oliveira-Biener et al. 2010; Schubart and Aichinger 2014).

*Pilumnus* has been extensively represented in scientific studies since the 18^th^ century (Linnaeus 1761; Leach 1816; Latreille 1825; Rathbun 1898). Various aspects of its members biology have been targeted, including ecology, reproduction, and larval development (Wear 1967; Bosa and Masunari 2000; Litulo 2005). Recently, evolutionary relationships within the genus have become a subject of investigations in several regions of the world by varied approaches. Using molecular data, Oliveira-Biener et al. (2010) accessed 141 crabs assigned to *Pilumnus* from the Mediterranean Sea and adjacent Atlantic waters, concluding that specimens identified as *Pilumnus hirtellus* (Linnaeus, 1761) belonged to three different lineages. Schubart and Aichinger (2014) analysed the Mediterranean *Pilumnus* but with a focus determining evolutionary units and possible hybridisations. Marin (2018) explored the taxonomic identity of Black Sea *Pilumnus* combining morphological and molecular data. Also working with a species from the Black Sea, *Pilumnus spinulosus* Kessler, 1861, Baytaşoğlu et al. (2023) examined different aspects of this species and used molecular data to help in the identification of the specimens. Finally, with a clear focus on the phylogeny of *Pilumnus*, Fahimi et al. (2021) investigated the taxonomy and the phylogenetic relationships among the species of *Pilumnus* in the Persian Gulf and the Gulf of Oman.

Despite previous studies, the understanding of phylogenetic relationships among species of *Pilumnus* is incomplete. One significant gap concerns species from the western Atlantic. Numerous studies have contributed to understanding diversity of *Pilumnus* along the American coasts (Milne-Edwards 1880; Rathbun 1930; Bookhout and Costlow 1979; Magalhães and Felder 2019). These have largely addressed the taxonomic status of congeners, though some have furthered systematic understanding of the genus either on the basis of morphological data alone (Spivak and Rodríguez 2002) or with a combination of morphological and molecular data (Magalhães et al. 2021). Nevertheless, no studies have focused primarily on the phylogenetic systematics of the western Atlantic species of *Pilumnus* or on the full set of North and South American congeners.

Nineteen species are currently described for western Atlantic waters (Rathbun 1930; Ng et al. 2008; Magalhães and Felder 2019): *Pilumnus caribaeus* Desbonne, in Desbonne & Schramm, 1867, *Pilumnus dasypodus* Kingsley, 1879, *Pilumnus diomedeae* Rathbun, 1894, *Pilumnus floridanus* Stimpson, 1871, *Pilumnus gemmatus* Stimpson, 1860, *Pilumnus gracilipes* A. Milne-Edwards, 1880, *Pilumnus holosericus* Rathbun, 1898, *Pilumnus lacteus* Stimpson, 1871, *Pilumnus longleyi* Rathbun, 1930, *Pilumnus mantelattoi* Magalhães & Felder, 2019, *Pilumnus marshi* Rathbun, 1901, *Pilumnus miersii* A. Milne-Edwards, 1880, *Pilumnus nudimanus* Rathbun, 1901, *Pilumnus pannosus* Rathbun, 1896, *Pilumnus quoii* H. Milne Edwards, 1834, *Pilumnus reticulatus* Stimpson, 1860, *Pilumnus sayi* Rathbun, 1897, *Pilumnus spinosissimus* Rathbun, 1898, and *Pilumnus vinaceus* A. Milne-Edwards, 1880. Thus, the high species diversity of this region makes it a key area for revisiting the taxonomy, systematics, and phylogenetics of the genus *Pilumnus*.

The present work undertakes a molecular phylogenetic study, augmenting and re-evaluating the outcomes of previous studies in which strong morphological similarities between species may mask species separations or variable morphologies may cause overestimation of diversity (Lefébure et al. 2006). The augmenting of previous morphologically based studies with molecular tools has proven to be effective in resolving phylogenetic and taxonomic issues within varied decapod crustacean groups (e.g., Schubart et al. 2000a; Munasinghe et al. 2003; Robles et al. 2007; Tsang et al. 2014; Magalhães et al. 2016; Terossi et al. 2017; Oliveira-Rogeri et al. 2023). We here extend such studies to members of the genus *Pilumnus* from the western Atlantic.

## ﻿Materials and methods

### ﻿Sampling

Tissue samples were obtained from both fresh specimens collected during the study and sequence-quality preserved material deposited in various crustacean collections. Fresh specimens were fixed in 80% ethyl alcohol and deposited at the Coleção de Crustáceos do Departamento de Biologia (**CCDB**) from the Faculdade de Filosofia, Ciências e Letras de Ribeirão Preto, Universidade de São Paulo (**FFCLRP/USP**).

Materials from decapod collections other than CCDB were accessed through visits, donations, and loans. These institutions included: Museu Nacional da Universidade Federal do Rio de Janeiro (**MNRJ**), Rio de Janeiro, Rio de Janeiro, Brazil; Museu de Zoologia da Universidade de São Paulo (**MZUSP**), São Paulo, São Paulo, Brazil; Museu Oceanografico Petronio Alves Coelho (**MOUFPE**), Universidade Federal do Pernambuco (**UFPE**), Recife, Pernambuco, Brazil; American Museum of Natural History (**AMNH**), New York, New York, United States; Muséum national d’Histoire naturelle (**MNHN**), Paris, France; United States National Museum (**USNM**), Washington D.C., United States; University of Louisiana-Lafayette Zoological collection (**ULLZ**), Lafayette, Louisiana, United States (the latter collection now incorporated into the collection of **USNM**).

### ﻿Marker selection

For the molecular analyses, two mitochondrial gene fragments were sequenced: 16S rRNA (16S) and Cytochrome Oxidase Subunit I (COI). The former is a gene with conserved regions and a slow evolutionary rate, widely used in phylogenetic reconstructions and taxonomic status verification (Schubart et al. 2000a; Mantelatto et al. 2009; Lai et al. 2011; Oliveira-Rogeri et al. 2023). The latter gene has a faster evolutionary rate, allowing the assessment to population structures (Moritz et al. 1987; Negri et al. 2012). Both mitochondrial genes have been extensively used in systematic studies of various invertebrate groups (Lefébure et al. 2006; Feng et al. 2011; Thoma et al. 2013). For decapod crustaceans, these genes have also been used in global projects, such as the Barcoding (Costa et al. 2007; Terossi and Mantelatto 2012; Silva et al. 2012) and the Tree of Life (Buhay and Crandall 2008; Bracken-Grissom et al. 2014), which focus on species delimitation and the elucidation of evolutionary relationships among different decapod crustacean groups.

### ﻿DNA extraction, amplification, and sequencing

Genomic DNA was extracted from muscle tissue, generally following protocols described by Schubart et al. (2000b), Mantelatto et al. (2006) and Robles et al. (2007), with specific adaptations (adjustments in the concentration of DNA sample and annealing temperature) for the present material. After extraction, DNA concentration of the samples was measured using the NanoDrop2000/2000c spectrophotometer (Thermo Scientific, Waltham, Massachusetts, USA).

Samples of DNA extraction products were amplified using Polymerase Chain Reaction (PCR) techniques on a Veriti thermal cycler system (Applied Biosystems, Foster City, California, USA), with primers as listed in Table 1. Steps and parameters of thermal cycles were as follows: initial denaturation at 94–95 °C for 5 min; for pairing, 35–40 cycles at 94–95 °C for 45 s, at 42–48 °C for 45 s, at 72 °C for 1 min; final extension at 72 °C for 5 min. Results were examined using electrophoresis techniques in 1.5% agarose gel and photographed with C-7070 Olympus (Tokyo, Japan) and SX520 Canon (Tokyo, Japan) digital cameras at UV M20 UVP and UV 302 Kasvi transilluminators. PCR products were purified using the SureClean Plus kit (Bioline, London, United Kingdom), following the manufacturer’s protocol.

**Table 1.** Primers used for DNA amplification.

Gene	Primer	Sequence	Reference
16S	1472 (H2)	5’-AGATAGAAACCAACCTGG-3’	Crandall and Fitzpatrick (1996)
	16L2 (L2)	5’-TGCCTGTTTATCAAAAACAT-3’	Schubart et al. (2000b)
COI	COH6	5’-TADACTTCDGGRTGDCCAAARAAYCA-3’	Schubart and Huber (2006)
	COL6b	5’-ACAAAATCATAAAGATATYGG-3’	Schubart and Huber (2006)

Purified samples were sent for DNA sequencing on ABI Prism 3100 Genetic Analyzer automated sequencers (Applied Biosystems, Foster City, CA, USA) at the Laboratório Multiusuário Centralizado para Sequenciamento de DNA em Larga Escala e Análise de Expressão Gênica, Departamento de Tecnologia, Faculdade de Ciências Agrárias and Veterinárias de Jaboticabal, Universidade Estadual Paulista (UNESP). The sequencing reaction was conducted with a total volume of 20 μL, following Schubart et al. (2000b) and Pileggi and Mantelatto (2010).

### ﻿Sequence editing and alignment

Forward and reverse strands for the 16S and COI mitochondrial gene fragments were generated and assembled to improve the quality of sequences. Edition and generation of consensus sequences were conducted using BIOEDIT 7.2.6 software (Hall 2005). All consensus sequences were submitted to the Basic Local Alignment Search Tool (BLAST) to compare them with the GenBank database, available at NCBI website (https://www.ncbi.nlm.nih.gov). Subsequently, sequences were aligned using MAFFT v. 7 (Katoh and Toh 2008) (http://mafft.cbrc.jp/alignment/server/). Sequences from protein-coding genes (e.g., COI) were translated to verify the presence of pseudogenes by checking the stop codons in the three different reading frames.

Sequences of 24 species from different zoogeographic regions were included in the analyses: *Pilumnus caribaeus*, *P. dasypodus*, *P. floridanus*, *P. gemmatus*, *P. holosericus*, *P. lacteus*, *P. longleyi*, *P. mantelattoi*, *P. marshi*, *P. nudimanus*, *P. pannosus*, *P. reticulatus*, *P. sayi* and *P. vinaceus* from the western Atlantic; *P. stebbingi* Capart, 1951 from the eastern Atlantic; *P. incanus* (Forskål, 1775), *P. longicornis* Hilgendorf, 1879, *P. minutus* De Haan, 1835, *P. orbitospinis* Rathbun, 1911, *P. persicus* Fahimi, Zolgharnein, Keykhosravi & Naderloo, 2021 and *P. savignyi* Heller, 1860 from the Indo-west Pacific; *P. tahitensis* de Man, 1890 from the central Pacific; *P. fernandezi* Garth, 1973 from the eastern Pacific; *P. hirtellus*, *P. spinifer* H. Milne Edwards, 1834, *P. spinulosus* and *P. villosissimus* (Rafinesque, 1814) from the Mediterranean Sea/North Sea. Most sequences accessed were new data generated by the present study, but our analyses also included sequences of other species of *Pilumnus* deposited in GenBank to expand the scope of our phylogenetic contextualisation. As outgroups, GenBank sequences of the following species were incorporated: *Chaceon ramosae* Manning, Tavares & Albuquerque, 1989, *Dracryopilumnus rathbunae* Balss, 1932, *Dairoides kusei* (Sakai, 1938), *Eriphia gonagra* (Fabricius, 1781), *Eupilumnus africanus* (A. Milne-Edwards, 1867) and *Menippe nodifrons* Stimpson, 1859. Genetic vouchers were deposited in the respective collections of origin, as indicated in Table 2.

**Table 2.** Specimens for which genetic sequences were included in genetic distance and phylogenetic analyses with their respective voucher, collection locality and GenBank accession numbers for each gene. For sequences not generated by the present study, the reference where first published is included in the table. (-) sequence of the correspondent gene is not available for that specimen.

Species and voucher	Locality	GenBank accession number		Reference
		COI	16S	
*Pilumnus caribaeus* Desbonne in Desbonne & Schramm, 1867				
ULLZ 14342	Florida, United States	MF504086	MF504042	Present study
ULLZ 11140	Belize	–	MF504045	Present study
MNHN-IU-2013-14497	Guadeloupe	MF504082	MF504043	Present study
ULLZ 13393	Panama	MF504087	MF504044	Present study
MNHN-IU-2014-9442	French Guiana	MF504088	–	Present study
MNHN-IU-2014-18494	French Guiana	MF504084	–	Present study
UFPE 15331	Pernambuco, Brazil	MF504094	–	Present study
UFPE 15329	Pernambuco, Brazil	MF504085	–	Present study
MNRJ 17249	Rio de Janeiro, Brazil	MF504092	–	Present study
CCDB 3615	São Paulo, Brazil	MF504079	–	Present study
CCDB 3615	São Paulo, Brazil	MF504089	MF504041	Present study
CCDB 3615	São Paulo, Brazil	MF504090	–	Present study
CCDB 3717	São Paulo, Brazil	MF504080	–	Present study
CCDB 3717	São Paulo, Brazil	MF504083	–	Present study
CCDB 5180	São Paulo, Brazil	MF504093	–	Present study
CCDB 5384	Brazil	MF504091	–	Present study
*Pilumnus dasypodus* Kingsley, 1879				
ULLZ 17141	Florida, United States	MF504095	–	Present study
AMNH 17848	Florida, United States	MF504101	–	Present study
ULLZ 16263	British Virgin Islands	MF504098	MF504046	Present study
ULLZ 4346	British Virgin Islands	MF504102	–	Present study
USNM 1277893	Belize	MF504096	–	Present study
MNHN-IU-2014-7412	Guadeloupe	MF504100	MF504048	Present study
CCDB 3550	Panama	MF504097	MF504047	Present study
MZUSP 29171	Bahia, Brazil	MF504099	–	Present study
*Pilumnus fernandezi* Garth, 1973				
ULLZ 12000	Panama	MF504103	MF504049	Present study
*Pilumnus floridanus* Stimpson, 1871				
ULLZ 12563	Belize	MF504105	MF504051	Present study
ULLZ 14354	Gulf of Mexico	MF504106	–	Present study
ULLZ 7343	Gulf of Mexico	–	EU863403	Thoma et al. (2009)
ULLZ 7317	Mexico	–	HM637980	Lai et al. (2014)
MNHN-IU-2013-2502	French Guiana	MF504108	–	Present study
MNRJ 17257	Rio de Janeiro, Brazil	MF504107	–	Present study
CCDB 5919	São Paulo, Brazil	MF504104	MF504050	Present study
*Pilumnus gemmatus* Stimpson, 1860				
MNHN-IU-2013-14558	Guadeloupe	MF504110	MF504052	Present study
ULLZ 13462	Panama	MF504109	MF504053	Present study
*Pilumnus hirtellus* (Linnaeus, 1761)				
MT03177	North Sea	KT209207	–	Raupach et al. (2015)
USNM 1277896	France	MF504111	MF504054	Present study
–	France	–	AM946023	Bui et al. (2008)
–	Portugal	HG328360	–	Schubart and Aichinger (2014)
*Pilumnus holosericus* Rathbun, 1898				
ULLZ 9921	Belize	MF504114	–	Present study
CCDB 3552	Panama	MF504112	–	Present study
ULLZ 13578	Panama	MF504113	MF504055	Present study
*Pilumnus incanus* (Forskål, 1775)				
–	Iran	MN545470	MN545464	Fahimi et al. (2021)
*Pilumnus lacteus* Stimpson, 1871				
ULLZ 15465	Belize	MF504115	MF504056	Present study
Pilumnus aff. lacteus				
MNHN-IU-2013-16321	Guadeloupe	MF504144	MF504071	Present study
*Pilumnus longicornis* Hilgendorf, 1878				
USNM 1277904	Iran	MF504116	–	Present study
–	Iran	MN545473	MN545461	Fahimi et al. (2021)
–	–	–	KJ132612	–
*Pilumnus longleyi* Rathbun, 1930				
ULLZ 11154	Belize	MF504117	MF504057	Present study
*Pilumnus mantelattoi* Magalhães & Felder, 2019				
ULLZ 10033	Belize	MF504143	–	Present study
*Pilumnus marshi* Rathbun, 1901				
ULLZ 17643	Belize	MF504118	MF504059	Present study
MNHN-IU-2014-7461	Guadeloupe	MF504119	MF504058	Present study
*Pilumnus minutus* De Haan, 1835				
USNM 1277854	South Korea	MF504120	MF504060	Present study
*Pilumnus nudimanus* Rathbun, 1901				
ULLZ 13466	Panama	MF504121	MF504061	Present study
*Pilumnus orbitospinis* Rathbun, 1911				
USNM 120711	Japan	MF504122	–	Present study
*Pilumnus pannosus* Rathbun, 1986				
ULLZ 11213	Texas, United States	MF504123	–	Present study
ULLZ 8637	Florida, United States	MF504124	MF504062	Present study
ULLZ 8373	Florida, United States	MF504125	MF504063	Present study
USNM 1277859	Panama	MF504126	–	Present study
*Pilumnus persicus* Fahimi, Zolgharnein, Keykhosravi & Naderloo, 2021				
–	Iran	MN545471	MN545463	Fahimi et al. (2021)
*Pilumnus reticulatus* Stimpson, 1860				
ULLZ 12562	Belize	MF504140	–	Present study
ULLZ 13589	Panama	MF504141	MF504070	Present study
UFPE 13508	Pernambuco, Brazil	MF504127	–	Present study
MNRJ 21463	Rio de Janeiro, Brazil	MF504134	–	Present study
CCDB 3617	São Paulo, Brazil	MF504142	MF504065	Present study
CCDB 3617	São Paulo, Brazil	MF504129	MF504066	Present study
CCDB 3617	São Paulo, Brazil	MF504139	–	Present study
CCDB 5391	São Paulo, Brazil	MF504135	–	Present study
CCDB 5391	São Paulo, Brazil	MF504137	–	Present study
CCDB 4323	São Paulo, Brazil	MF504128	–	Present study
CCDB 4323	São Paulo, Brazil	MF504136	–	Present study
MZUSP 23490	Santa Catarina, Brazil	MF504133	–	Present study
CCDB 5379	Argentina	MF504130	MF504067	Present study
CCDB 5379	Argentina	MF504132	MF504068	Present study
CCDB 5379	Argentina	MF504131	MF504069	Present study
CCDB 227	Brazil	MF504138	–	Present study
*Pilumnus sayi* Rathbun, 1897				
ULLZ 14405	Florida, United States	–	MF504072	Present study
ULLZ 6938	Florida, United States	–	GU144435	–
*Pilumnus savignyi* Heller, 1860				
–	Iran	MN545472	MN545460	Fahimi et al. (2021)
*Pilumnus spinifer* H. Milne Edwards, 1834				
–	Croatia	HG328359	–	Schubart and Aichinger (2014)
USNM 1277963	Tunisia	MF504145	–	Present study
*Pilumnus spinulosus* Kessler, 1861				
–	Bulgaria	HG328358	–	Schubart and Aichinger (2014)
–	Turkey	MW411952	–	Baytaşoğlu et al. (2023)
–	Turkey	ON878102	–	Baytaşoğlu et al. (2023)
*Pilumnus stebbingi* Capart, 1951				
USNM 1277872	Equatorial Guinea	MF504146	–	Present study
*Pilumnus tahitensis* de Man, 1890				
USNM 266710	French Polynesia	MF504147	–	Present study
*Pilumnus vespertilio* (Fabricius, 1793)				
–	Taiwan	–	KJ132627	Tsang et al. (2014)
–	Philippines	–	FJ548952	Lai et al. (2009)
*Pilumnus villosissimus* (Rafinesque, 1814)				
–	Spain	HG328357	–	Schubart and Aichinger (2014)
*Pilumnus vinaceus* A. Milne-Edwards, 1880				
ULLZ 4432	United States	MF504155	MF504076	Present study
CCDB 5383	Ceará, Brazil	MF504152	–	Present study
CCDB 5383	Ceará, Brazil	MF504154	–	Present study
CCDB 5383	Ceará, Brazil	MF504156	–	Present study
MZUSP 32326	Espírito Santo, Brazil	MF504153	–	Present study
MNRJ 18367	Rio de Janeiro, Brazil	MF504151	–	Present study
CCDB 5382	São Paulo, Brazil	MF504148	MF504075	Present study
CCDB 4593	São Paulo, Brazil	MF504150	–	Present study
CCDB 4598	São Paulo, Brazil	–	MF504078	Present study
MZUSP 12570	Brazil	MF504149	–	Present study
Outgroups				
*Chaceon ramosae* Manning, Tavares & Albuquerque, 1989				
–	Brazil	KC676779	KC676756	Mantelatto et al. (2014)
*Dacryopilumnus rathbunae* Balss, 1932				
ZRC 1999.1291	Japan	HM638027	HM637971	Lai et al. (2014)
*Dairoides kusei* (Sakai, 1938)				
ULLZ 9183	Hawaii, United States	HM638030	HM637979	Lai et al. (2014)
*Eriphia gonagra* (Fabricius, 1781)				
ULLZ 5463	Florida, United States	HM638035	HM637964	Lai et al. (2014)
*Eupilumnus africanus* (A. Milne-Edwards, 1867)				
ULLZ 11966	Green Cape	KC771025	KC771007	Lai et al. (2014)
*Menippe nodifrons* Stimpson, 1859				
ULLZ 4351	Mexico	HM638050	HM637975	Lai et al. (2014)

### ﻿Genetic distance analyses

Genetic distance parameters were used to address systematic issues, as applied by previous authors in taxonomic studies of various crustacean taxa (Weber et al. 2000; Munasinghe et al. 2003; Ocampo et al. 2013). The presence of patterns in nucleotide differences between DNA sequences, both intra- and interspecific, facilitated the determination of genetic similarity boundaries. These values were instrumental in evaluating whether individual specimens should be classified as the same species or different species. For western Atlantic members of *Pilumnus*, genetic distance analyses were performed using MEGA 10.0.5 (Nei and Kumar 2000; Tamura et al. 2004; Kumar et al. 2018). Genetic distance values were calculated using p-distance, since it avoids over-parameterisation and there is no need to use complex distance measurements when studying closely related sequences (Nei and Kumar 2000; Collins et al. 2012). Two genetic distance matrices were constructed, one for each gene analysed in this present study.

### ﻿Phylogenetic analyses

To infer evolutionary relationships within *Pilumnus*, two phylogenetic hypotheses were constructed: one based on Maximum Likelihood (ML) and one on Bayesian Inference (BI). The data used for these hypotheses consisted of genetic sequences from two mitochondrial genes: Cytochrome Oxidase Subunit I (COI) and 16S rRNA (16S). The final alignment included sequences with a total of 1247 base pairs (660 from COI and 587 from 16S). We used the online software IQ-TREE (http://www.iqtree.org/) (Nguyen et al. 2015) to construct the ML hypothesis. The best evolutionary model for generation of the phylogenetic tree was determined using the Model Finder present at IQ-Tree. Based on the Bayesian Information Criteria (BIC), the optimal model for our dataset was GTR+F+I+G4. Branch supports were evaluated using the bootstrapping (bs) method. For BI hypothesis, we used MEGA 10.0.5 (Nei and Kumar 2000; Tamura et al. 2004; Kumar et al. 2018) to identify the best evolutionary models for each gene. Model selection was also based on BIC values. For COI, the best model was GTR+G+I, and for 16S, it was TN93+G. Three separate analyses were run using BEAST 2.5 (Bouckaert et al. 2019). Analysis specifications were defined in BEAUTI 2 (Bouckaert et al. 2019), with each analysis consisting of 20 million generations. The results of each analysis were summarised using TREEANNOTATOR v. 2.6.2 (Drummond and Rambaut 2007). The outcomes of the three analyses were combined using LOGCOMBINER v. 2.6.6 (Drummond and Rambaut 2007), which facilitated the generation of a final consensus phylogenetic hypothesis. Branch support was evaluated based on posterior probability (pp) values.

### ﻿Estimation of divergence time

In order to estimate the divergence time of each group, our BI phylogenetic hypothesis included a molecular clock. Based on the analysis specifications defined in BEAUTI 2, we selected the Relaxed Clock Log Normal model as our clock model. To calibrate the tree, we included fossil data, specifically from *Galenopsis* sp. A. Milne-Edwards (1865) from the Middle Eocene, considered a stem group of Pilumnidae Samouelle 1819 (Tsang et al. 2014).

### ﻿Species delimitation

We employed two species delimitation methods to determine if each currently recognised species within Pilumnidae represents a single Molecular Operational Taxonomic Unit (MOTU). As models for species delimitation, we used the methods Poisson Tree Processes (PTP) and General Mixed Yule Coalescent (GMYC) (Zhang et al. 2013), both of which have been applied in species delimitation analyses for other decapod crustaceans (Crivellaro et al. 2018; Ramirez et al. 2021; Mantelatto et al. 2023). PTP uses a phylogenetic tree to infer possible speciation events based on the number of substitutions. GMYC also models speciation events based on substitutions but requires a time-calibrated phylogenetic tree. These methods were applied on the BI phylogenetic tree using The Exelixis Lab online servers (https://species.h-its.org/ptp/ and https://species.h-its.org/gmyc/) (Zhang et al. 2013). The server provides a Bayesian implementation for PTP model (bTPT), which attributes Bayesian support for the delimitations. To PTP and bPTP, we ran 500,000 generations with a burn-in of 0.1. In the case of GMYC method, the server provides a backend which runs the original R implementation of the model (Fujisawa and Barraclough 2013).

## ﻿Results

### ﻿Genetic distance analyses

**Figure 1.**
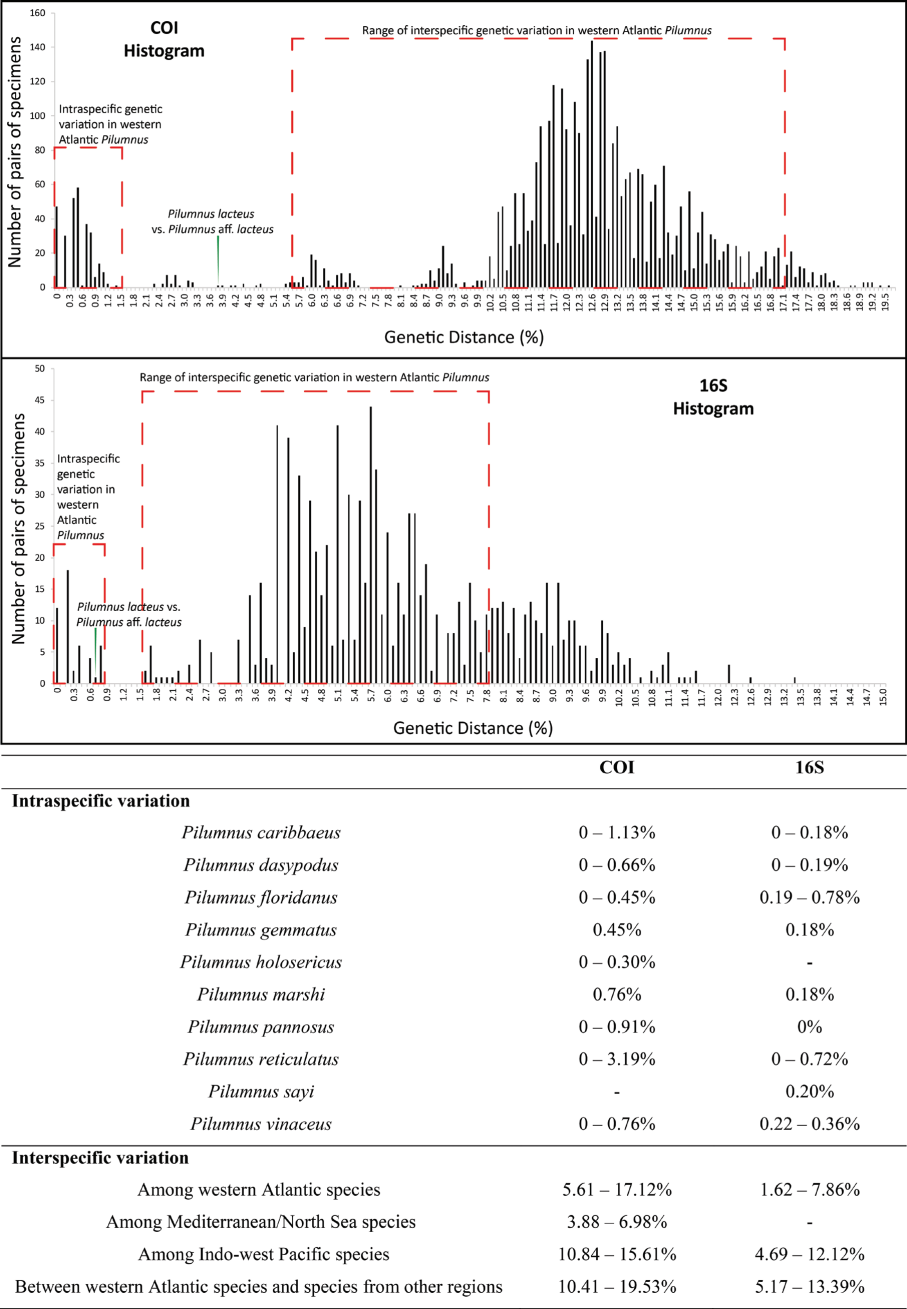
Genetic distances among species of *Pilumnus* based on COI and 16S genetic sequences. On the top, two histograms (one for each gene) indicate the frequencies of occurrence of each value of genetic distance in the analysed dataset. On the bottom, a summary of some intra- and interspecific genetic divergences is presented, with the intervals representing minimum and maximum values of genetic distance. (-) indicates that no more than one genetic sequence was available.

Genetic distances were estimated for all accessed genetic sequences, and histograms exhibiting the divergence detected for each gene were created (Fig. 1), with a summary of genetic distance values. The genetic distance values based on COI gene sequences indicated a clear genetic distinction among each currently recognised species, as shown in the histogram. Among western Atlantic congeners, intraspecific genetic distance values varied from 0 to 3.19%. For every species analysed with more than two specimens, the minimum intraspecific genetic distance was 0% (i.e., *P. caribaeus*, *P. dasypodus*, *P. floridanus*, *P. holosericus*, *P. pannosus*, *P. reticulatus*, and *P. reticulatus*). The maximum intraspecific genetic distance was observed in *P. reticulatus* (= 3.19%), which was significantly higher than the maximum values obtained for other species, such as *P. caribaeus* (= 1.13%) and *P. pannosus* (= 0.91%).

The range of interspecific genetic divergence varied among different geographic regions. For the western Atlantic, represented by ten species in our analyses, divergence ranged from 5.61% to 17.12%. For the Mediterranean/North Sea, represented by four species, and Indo-west Pacific, represented by seven species, the ranges of variation were 3.88–6.98% and 10.84–15.61%, respectively. Comparing western Atlantic species with species from the other regions, the divergence ranged from 10.41% to 19.53%. As eastern Atlantic, and eastern and central Pacific regions were each represented by only one species, interspecific variation could not be evaluated.

For the 16S gene, the histogram of genetic distance values showed a gap between intra- and interspecific values, again indicating a clear separation between described species (Fig. 1). Intraspecific divergence values varied from 0% to 0.78%. The minimum intraspecific genetic distance was 0% for several species (i.e., *P. caribaeus*, *P. dasypodus*, *P. pannosus*, and *P. reticulatus*). The maximum intraspecific values were observed for *P. floridanus* (= 0.78%) and *P. reticulatus* (= 0.72%).

Regarding interspecific variation by biogeographic region, western Atlantic species showed divergence ranging from 1.62% to 7.86% (Fig. 1), while Indo-west Pacific species exhibited variation from 4.69% to 12.12%. When comparing western Atlantic species with those from other regions, divergence values ranged from 5.17% to 13.39%. For the 16S gene, we had access to only one species from the Mediterranean/North Sea, which precluded an assessment of regional genetic divergence there.

### ﻿Phylogenetic analyses and species delimitation

**Figure 2.**
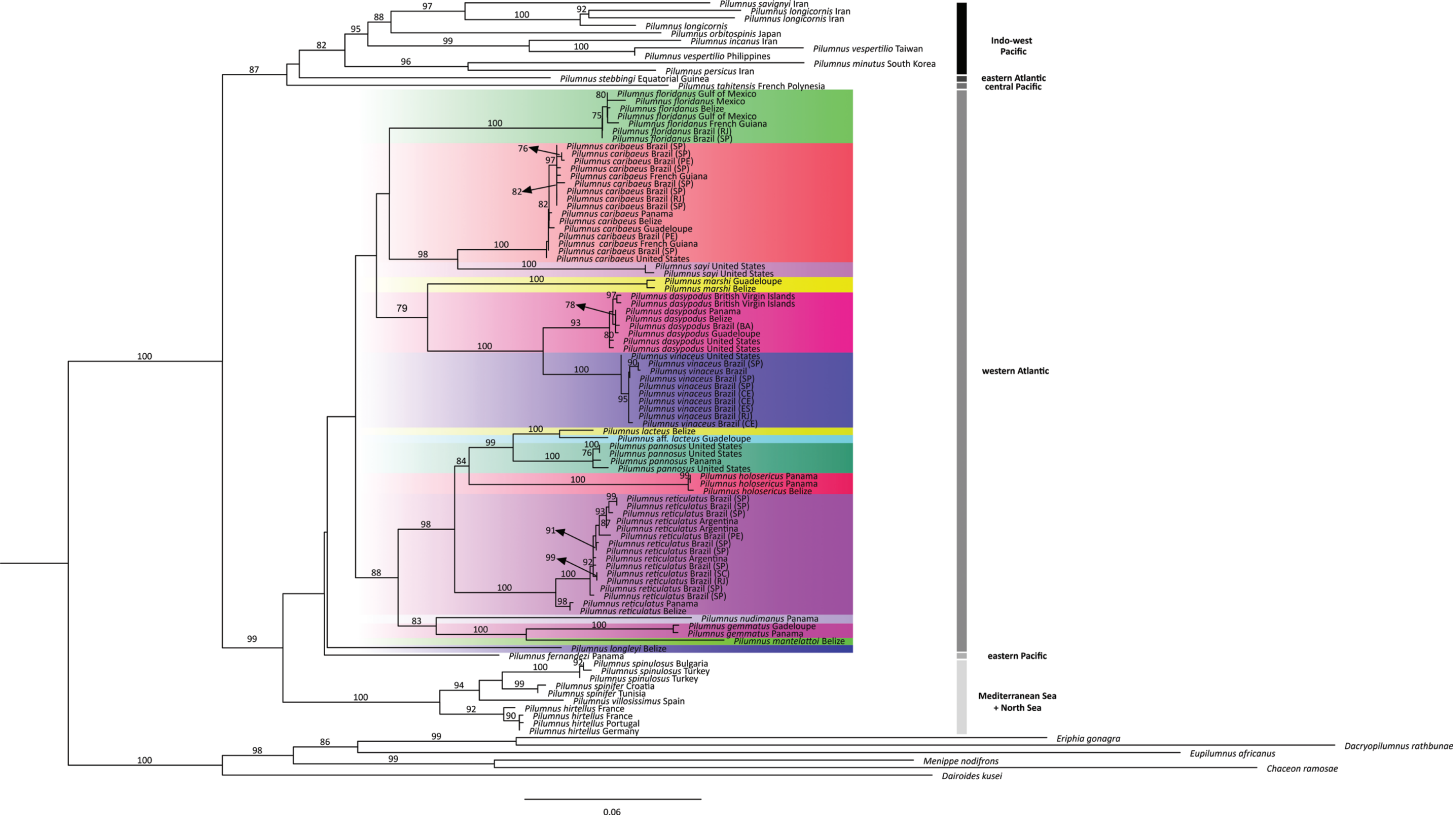
Phylogenetic hypothesis built using sequences of COI and 16S genes based on Maximum Likelihood. Brach supports are presented as bootstrap values; those below 75 are not presented. On the right of the tree, we included the biogeographic regions of each specimen. BA, Bahia; CE, Ceará; ES, Espírito Santo; PE, Pernambuco; RJ, Rio de Janeiro; SC, Santa Catarina; SP, São Paulo.

**Figure 3.**
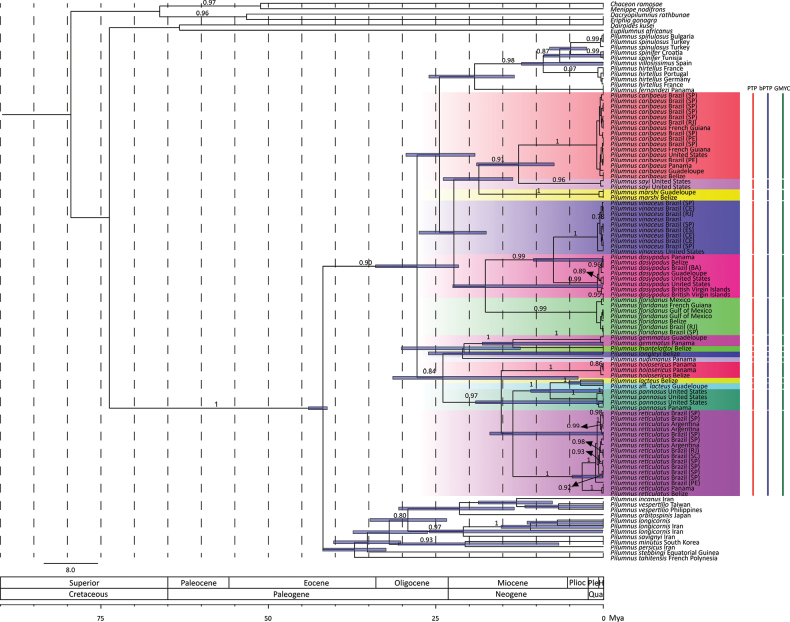
Phylogenetic hypothesis built using sequences of COI and 16S genes based on Bayesian Inference. Brach supports are presented as posterior probability values; those below 0.75 are not presented. Purple node bars indicate the confidence interval (highest posterior density – 95%) of the age of each node. Below the tree, a rule presents the geological time. On the right of the tree, is presented the result of the species delimitation methods with the 16 Molecular Operational Taxonomic Units suggested by both methods. PTP, Poisson Tree Process; bPTP, Bayesian implementation for Poisson Tree Process; GMYC, General Mixed Yule Coalescent; Plioc, Pliocene; Ple, Pleistocene; H, Holocene; Qua, Quaternary; Mya, Million years ago; BA, Bahia; CE, Ceará; ES, Espírito Santo; PE, Pernambuco; RJ, Rio de Janeiro; SC, Santa Catarina; SP, São Paulo.

Whether based on ML or BI (Figs 2, 3, respectively), our phylogenetic hypotheses resolve *Pilumnus* as a monophyletic group with maximum support in both trees (bs = 100; pp = 1). The relationships among species within *Pilumnus*, however, varied between the two hypotheses, in some cases congruent with current taxonomy and previously proposed relationships, in others inconsistent with them.

Available American species of *Pilumnus*, including those from both the western Atlantic and eastern Pacific, when combined with Mediterranean/North Sea species formed a well-supported group in both ML and BI trees (bs = 99; pp = 0.90) in our analyses (Figs 2, 3). A grouping restricted to only the American species formed a monophyletic lineage in the ML tree, although only with low support. Similarly, western Atlantic representatives formed a clade with low support in the ML tree. In contrast, the BI hypothesis suggests that some American species could be more closely related to Mediterranean/North Sea species than to other American species, though this relationship had low support. Mediterranean/North Sea species formed a well-supported clade in both trees (bs = 100; pp = 0.98). Indo-west Pacific species formed a clade with moderate support in the ML tree (bs = 82), but with low support in the BI reconstruction. Among topologies with low support, the eastern Atlantic species *P. stebbingi* resolved as a sister group to the Indo-west Pacific clade, while the central Pacific species *P. tahitensis* was positioned as a sister to the *P. stebbingi* + Indo-west Pacific clade.

Within the western Atlantic group, *P. longleyi* resolved as the sister clade to all other species in ML reconstruction. Excluding *P. longleyi*, the remaining species were divided into two distinct lineages. The first lineage, with low support, included *P. caribaeus*, *P. dasypodus*, *P. floridanus*, *P. marshi*, *P. sayi*, and *P. vinaceus*. The second linage, with moderate support (bs = 88) grouped *P. gemmatus*, *P. holosericus*, *P. lacteus*, *P. mantelattoi*, *P. nudimanus*, *P. pannosus*, and *P. reticulatus*. In the BI hypothesis, these two lineages were also resolved, but with some variations as follow. The first lineage (including *P. caribaeus*, *P. dasypodus*, *P. floridanus*, *P. marshi*, *P. sayi*, and *P. vinaceus*) was positioned as a sister group to the Mediterranean/North Sea clade, although with low support. The second lineage, which included *P. longleyi* along with the previously mentioned species, had moderate support in the BI reconstruction (pp = 0.84).

The more restricted groups exhibited higher supports in both hypotheses, though some relationships varied between the two trees. *Pilumnus holosericus*, *P. lacteus*, *P. pannosus*, and *P. reticulatus* formed a monophyletic group with high support in both reconstructions (bs = 98; pp = 0.97). An even more restricted group, formed by *P. lacteus* and *P. pannosus*, was strongly supported (bs = 99; pp = 1). The ML tree also showed a close relationship among *P. gemmatus*, *P. mantelattoi*, and *P. nudimanus* (bs = 83). In the BI tree, *P. gemmatus* and *P. mantelattoi* were sister groups with high support, but *P. nudimanus* did not resolve as their sister taxon; instead, *P. longleyi* occupied that position, though with low support.

Two clear congruencies between both hypotheses include the close relationships between *P. dasypodus* and *P. vinaceus*, and between *P. caribaeus* and *P. sayi*. *P. dasypodus* was resolved as the sister taxon to *P. vinaceus* (bs = 100; pp = 0.99), while *P. caribaeus* as the sister species to *P. sayi* (bs = 98; pp = 0.91).

Monophyly could not be evaluated for *P. lacteus*, *P. longleyi*, *P. mantelattoi*, and *P. nudimanus* given limitation of those sequence data to single specimens of each species. For all cases in which a species was represented by multiple specimens, identified *a priori* by morphological characters in existing taxonomic literature, monophyly was confirmed. In both the ML and BI trees, all specimens assigned to the same species formed monophyletic groups with moderate to high support, as follow: *P. caribaeus* (bs = 100; pp = 1), *P. dasypodus* (bs = 100; pp = 0.99), *P. floridanus* (bs = 100; pp = 0.99), *P. gemmatus* (bs = 100; pp = 1), *P. holosericus* (bs = 100; 1), *P. marshi* (bs =100; pp = 1), *P. pannosus* (bs = 100; pp = 1), *P. reticulatus* (bs = 100; pp = 1), *P. sayi* (bs = 100; pp = 0.96) and *P. vinaceus* (bs = 100; pp = 1).

Species delimitation methods, PTP, bPTP, and GMYC, indicated 16 different MOTUs among the 14 genetically accessed western Atlantic species of *Pilumnus*. In addition to the 14 described species, the two additional MOTUs corresponded to a specimen of *Pilumnus* from Guadeloupe closely related to *P. lacteus* and another within *P. reticulatus* that was subdivided into two distinct lineages.

## ﻿Discussion

### ﻿Phylogeny and diversification of western Atlantic bristle crabs

The present study provides an unprecedented analysis of the phylogenetic relationships among species of the genus *Pilumnus* from the western Atlantic. While sequence-quality tissues for some species of the region were not available to us (namely, *P. diomedeae*, *P. gracilipes*, *P. miersii*, *P. quoii* and *P. spinosissimus*), genetic sequences from 14 species were included in the analyses, representing nearly 75% of the diversity of the genus in the western Atlantic. The evaluated specimens included material from coastlines ranging from United States to Argentina, thus covering North America, Central America (including the Caribbean Sea), and the South America.

Our phylogenetic hypotheses do not conclusively determine whether western Atlantic *Pilumnus* form a monophyletic group due to the lack of congruence between the hypotheses and the absence of some species in our analysis. In the BI tree, some western Atlantic species are topologically closer to Mediterranean/North Sea species than to other western Atlantic representatives. Conversely, in the ML tree, they form a clade, but with low support. The estimation of divergence time indicates that the clade comprising American (western Atlantic + eastern Pacific) and including Mediterranean/North Sea species originated at some timeframe during the Oligocene (from ca 33.9 to 23 Mya).

Our phylogenetic hypotheses suggest that western Atlantic species form two distinct lineages: one with low and another with moderate support. The low-supported lineage consists of six species (*P. caribaeus*, *P. dasypodus*, *P. floridanus*, *P. marshi*, *P. sayi*, and *P. vinaceus*). If valid, this linage’s diversification, according to our estimation of divergence time, started between the Late Oligocene and the Early Miocene (between 28 and 18 Mya). The moderate supported lineage includes at least seven species (*P. gemmatus*, *P. holosericus*, *P. lacteus*, *P. mantelattoi*, *P. nudimanus*, *P. pannosus*, and *P. reticulatus*). Although more supported than the first, accurate assumptions about the time of divergence for this lineage are difficult due to the excessively broad interval provided by the estimation.

The division of western Atlantic species in two lineages does not appear to have an obvious geographical basis, as both lineages include species from various and similar regions of the western Atlantic. Some of the species from both lineages even have wide distributions. For instance, specimens of *P. dasypodus* originate from the United States (North America), Panama (Central America), and Brazil (South America).

Our data also suggest that sympatry is extremely common, even among closely related species. For example, *Pilumnus dasypodus* and *P. vinaceus* coexist in northeastern Brazil, and the closely related *P. holosericus* and *P. lacteus* are found together in Belize. Indeed, this Central American country with a comparatively short coastline exemplifies what may be typical sympatry among species of *Pilumnus* in tropical western Atlantic waters, with at least ten species often sharing the same habitats (*P. caribaeus*, *P. dasypodus*, *P. floridanus*, *P. holosericus*, *P. lacteus*, *P. longleyi*, *P. mantelattoi*, *P. marshi*, *P. reticulatus*, and *P. vinaceus*). Other countries of the region, such as Panama, show similar patterns of high diversity and high sympatry, reinforcing the widespread theory of the high diversity of marine decapod crustaceans in the Caribbean Sea (De Grave and Anker 2017; Poupin 2018; Parasram et al. 2023).

### ﻿Biogeographical inferences

Although monophyly of eastern Atlantic *Pilumnus* remains to be confirmed, our analyses do suggest an intimate relationship between western Atlantic, eastern Pacific and Mediterranean/North Sea species, particularly when compared to Indo-west Pacific species. The clade comprising species from the three aforementioned biogeographic regions originated during in Oligocene. While diversification along the America coast started during this period (between Late Oligocene and Early Miocene), diversification in the Mediterranean Sea, according to our estimations, did not start until ca 12.5 Mya. This corresponds to the Middle Miocene, a time when the Mediterranean Sea was being influenced by the Atlantic biota (Bianchi et al. 2011). Hence, this geological context helps explain the close relationship between western Atlantic and Mediterranean species. Furthermore, the absence of a Panamanian Isthmus during the Oligocene and Miocene can explain the genetic proximity between eastern Pacific and western Atlantic species.

Due to limited sampling of eastern Pacific and eastern Atlantic waters in our study, we cannot confirm that the Mediterranean/North Sea and eastern Pacific species are the most closely related to the western Atlantic representatives. From the eastern Pacific, we included *P. fernandezi*, which appears as the sister group to the western Atlantic clade in ML tree and to the Mediterranean/North Sea clade in BI tree. From the eastern Atlantic, we included *P. stebbingi*, which in both analyses appears as sister group to the Indo-west Pacific clade.

Our study thus provides initial insights into the biogeography of *Pilumnus*, but with a bias to the western Atlantic region. A more robust evolutionary model, perhaps with more refined understanding of biogeographic processes at play in the separation of species groupings within the genus, will likely require full representation of western Atlantic congeners, broader sampling of geographically separated populations, more comprehensive representation of eastern Pacific and eastern Atlantic populations, and the use of additional genetic markers.

### ﻿Described species vs detected MOTUs

In at least two instances, our findings suggest that western Atlantic materials included in our analyses could represent possibly undescribed MOTUs. In one case, our phylogenetic hypotheses indicated two different lineages in *P. reticulatus*, and species delimitation methods suggested that these two lineages might correspond to two distinct species. This separation is supported by genetic divergence for the COI gene and geographic congruence. Comparing specimens of *P. reticulatus* from Caribbean Sea (Belize and Panama) and the South Atlantic (Brazil and Argentina), we detected genetic distance values higher than the intraspecific values observed among all other species, but lower than all interspecific values observed for the accessed species of *Pilumnus*. The variation between these two groups of *P. reticulatus* ranged from 2.27% to 3.30%. The maximum intraspecific genetic distance recorded within any other species was 1.13%. In contrast, the minimum interspecific value was 5.61%, when considering only western Atlantic species but 3.88% when considering the Mediterranean/North Sea species. The genetic distance between two lineages of *P. reticulatus* is therefore close to the divergence observed between two previously separated and described species of *Pilumnus*.

Considering that one lineage comprises two Caribbean specimens (from Panama and Belize, respectively) and the other contains the remaining specimens of *P. reticulatus* (all of them from Brazil), we hypothesise that there may be a marine geographical barrier in northern South America which hinders gene flow between the two lineages (Peres and Mantelatto 2020; Peres et al. 2022). A definitive conclusion regarding whether the two groups represent different species or just different populations of *P. reticulatus* requires a detailed morphological analysis of representatives from both lineages. This must also take into account the possible significance either lineage aligning with one of the several morphological “forms” of this species proposed by Rathbun (1930: 522–523), the names of these forms being derived from previously applied species names treated by her as junior synonyms. Such further studies might also further examine relationships to reported Pacific population of the species, including both the Gulf of California and Pacific side of the Panama Canal, as summarised by Abele and Kim (1989).

Another potentially undescribed MOTU corresponds to a specimen from Guadeloupe (MNHN-IU-2013-16321). In both phylogenetic hypotheses, this species appears as the sister group of *P. lacteus*. Genetic divergence between the Guadeloupe specimen and *P. lacteus* was 3.9% for the COI gene, and 0.7% for 16S. These values are above the maximum intraspecific genetic distance values registered among the western Atlantic accessed specimens (1.13% for COI, excluding *P. reticulatus*; 0.6% for 16S). Due to the close relationship between the Guadeloupe specimen and *P. lacteus*, we for now define this Guadeloupe specimen as Pilumnus aff. lacteus. Future investigations in progress, including thorough morphological analysis, may indicate if this P. aff. lacteus specimen represents a new species of the genus.

Our analyses also offer further molecular genetic basis for the separation of *P. vinaceus* from *P. dasypodus*, as was previously reported by Magalhães et al. (2021) and briefly addressed by Clark et al. (2025). The resurrection of *P. vinaceus* from its long synonymy with *P. dasypodus* was supported by molecular phylogenetic analyses of Magalhães et al. (2021) that distinguished Brazilian populations they assigned to *P. vinaceus* from what were otherwise regarded to be widely distributed western Atlantic populations of *P. dasypodus*. That separation is more robustly supported in the present analyses, for the first time with the addition of sequences for a northern hemisphere specimen of *P. vinaceus* (ULLZ 4432) from the western Gulf of Mexico (Texas). The importance of this is proximity of that site to the Florida Keys type locality for *P. vinaceus*, thus for the first time confirming molecular genetic identity of a specimen from this region with sequenced specimens from Brazil. Previously, the report of conspecifics from these widely separated localities had been based solely on subtle morphological characters, problematic even in separations of *P. dasypodus* from *P. vinaceus*. Morphological separations of these two species continue to be challenging, especially since the type of *P. dasypodus* is not extant. The “type” (YMP IZ 022640) reported by Magalhães et al. (2021) was misconstrued as such, being only a hypotype, referring to a published collection reported by Boone (1927) from Cuban waters.

## ﻿Conclusions

The present study helps to elucidate the phylogeny of *Pilumnus* within the western Atlantic. By analysing an informative number of specimens, we evaluated the phylogenetic relationships and the patterns of genetic divergence not only within the western Atlantic species, but also comparing them with species from other geographic regions. Notably, the analysis of genetic distances highlighted clear distinction among recognised species, with *P. reticulatus* exhibiting significant intraspecific divergence, suggesting the presence of two distinct lineages. Species delimitation methods identified 16 different MOTUs among the 14 genetically assessed western Atlantic species of *Pilumnus*, raising the possibility of existence of undescribed species. The findings underscore the high and likely underestimated diversity of marine decapod crustaceans, particularly within *Pilumnus* and provide insights into the biogeography. The close relationship between western Atlantic, eastern Pacific, and Mediterranean/North Sea species, and their divergence times suggest significant diversification events from the Late Oligocene to the Early Miocene. In summary, this study offers a significant step towards understanding the evolutionary patterns and processes which characterise the hairy crabs. It also evinces the potential for further discoveries and emphasises the importance of comprehensive taxonomic studies to reveal the true diversity within marine decapod crustaceans.

## ﻿Additional information

### Use of AI

No use of AI was reported.

### Author ORCIDs

Lucas Oliveira-Rogeri https://orcid.org/0009-0002-6686-6723

Tatiana Magalhães https://orcid.org/0000-0003-1573-3533

Darryl L. Felder https://orcid.org/0000-0001-7679-7712

Fernando L. Mantelatto https://orcid.org/0000-0002-8497-187X

### Data availability

All research data/code are available in the text. DNA sequences generated were deposited at GenBank (https://www.ncbi.nlm.nih.gov/genbank/) and their accession numbers are available on Table 2.
